# Comparison of Face Washing and Face Wiping Methods for Trachoma Control: A Pilot Study

**DOI:** 10.4269/ajtmh.19-0726

**Published:** 2020-02-10

**Authors:** Alexandra Czerniewska, Aalbertus Versteeg, Oumer Shafi, Gebeyehu Dumessa, Muluadam Abraham Aga, Anna Last, David MacLeod, Virginia Sarah, Sarity Dodson, Nebiyu Negussu, Birhanu Kenate Sori, Michael Kirumba, Adam Biran, Sandy Cairncross, Matthew J. Burton, Katie Greenland

**Affiliations:** 1 Department of Disease Control, London School of Hygiene and Tropical Medicine, London, United Kingdom;; 2 Clinical Research Department, London School of Hygiene and Tropical Medicine, London, United Kingdom;; 3 The Fred Hollows Foundation, Addis Ababa, Ethiopia;; 4 Department of Infectious Disease Epidemiology, London School of Hygiene and Tropical Medicine, London, United Kingdom;; 5 The Fred Hollows Foundation, London, United Kingdom;; 6 The Fred Hollows Foundation, Sydney, Australia;; 7 Federal Ministry of Health, Addis Ababa, Ethiopia;; 8 Oromia Regional Health Bureau, Addis Ababa, Ethiopia;; 9 Clinical Research Department, International Centre for Eye Health, London School of Hygiene and Tropical Medicine, London, United Kingdom;; 10 Cornea and External Eye Department, Moorfields Eye Hospital NHS Trust, London, United Kingdom

## Abstract

Eye-to-eye transmission of *Chlamydia trachomatis*, the causative agent of trachoma, may be plausibly interrupted if faces are kept free of ocular and nasal discharge. Between April and June 2018, 83 children aged 1–9 years with active trachoma were recruited from 62 households and allocated to a face cleaning protocol: face washing with water, face washing with water and soap, or face wiping. Faces were examined for the presence of ocular and nasal discharge, and swabs were taken from faces and hands to test for *C. trachomatis* at baseline, immediately post protocol, and after 1, 2, and 4 hours (washing protocols). Washing with soap was more effective at removing ocular discharge than either washing with water (89% and 27% of discharge removed, respectively, *P* = 0.003) or wiping with a hand (42%, *P* = 0.013). The reduction in prevalence of ocular discharge was sustained for at least four hours. The prevalence of *C. trachomatis* on face swabs was reduced by all washing protocols. The importance of soap should not be overlooked during facial cleanliness promotion.

## INTRODUCTION

Trachoma is the most common infectious cause of blindness worldwide.^1^ Children are the main reservoir of ocular infection with the bacterium *Chlamydia trachomatis*. This provokes chronic conjunctival inflammation, scarring, and in-turned eyelids, which can lead to irreversible corneal damage and sight loss.

Endemic trachoma is maintained through ongoing transmission. Improving facial cleaning practice across at-risk communities is a key pillar of the WHO’s strategy for trachoma elimination.^1^ *Chlamydia trachomatis* is probably transmitted through the transfer of ocular and nasal discharge of an infected person. Hypothesized *C. trachomatis* transmission routes, such as via the face or hands, contaminated objects, or mechanical vectors, are plausibly interrupted if faces are kept free of discharge.^1,2^

Although research has linked prevalence of disease symptoms with facial cleanliness,^3^ there are limited systematic data on the effectiveness of different face cleaning techniques or frequencies to reduce the risk of transmission.^4^ Health promotion programs consequently recommend an array of face washing messages and times.^4^ Most recommend soap use, but emphasis varies. The current study, conducted to inform the design of a large-scale face washing intervention, aimed to identify the most effective hygiene technique and frequency to minimize potential *C. trachomatis* transmission.

## METHODS

We conducted this study in the West Arsi Zone of Oromia, Ethiopia, between April and June 2018. The study was nested within a cross-sectional study conducted in 247 randomly selected households with at least one child aged 1–9 years with clinically diagnosed trachomatous inflammation, follicular (TF) or trachomatous inflammation, intense (TI).^5,6^ Children aged 1–9 years with TF/TI were eligible for inclusion in this sub-study.

We defined ocular discharge as the presence of clear or cloudy fluid, or dry matter, on the lid margin or lid (including the corners) and nasal discharge as the presence of wet or dry discharge visible outside the nostril nares.^7^ At baseline, faces were examined for the presence of ocular and nasal discharge. A trained field-worker collected sterile, dacron swab samples from 1) the eye (to determine *C. trachomatis* infection status), 2) the face, and 3) the hands. As per the Standard Operating Procedure, ocular swabs were wiped four times across the (everted) left upper tarsal conjunctival surface. Face and hand swabs were premoistened with sucrose phosphate buffer (2SP) and systematically rubbed with moderate, consistent pressure, tracing a line under the right eye, across the right cheek and under the nare of the right nostril (face), or across palms and backs of both hands, finger pads, and in between each finger (hands). One air control swab was randomly collected for each 50 samples to evaluate field and laboratory contamination. Gloves were changed between swabs. Swabs were stored immediately in microtubes containing 500 μL of 2SP transport medium on ice packs, transferred to a −20°C freezer within 8 hours of collection and then to a −80°C freezer within 1 week, until testing.

**Figure 1.**
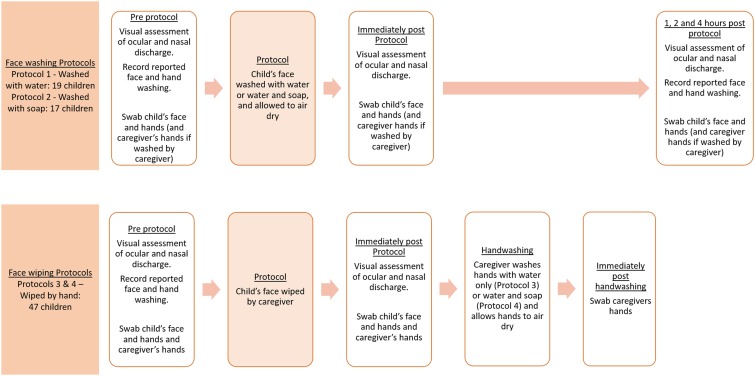
Schematic illustrating the face washing and face wiping protocols. This figure appears in color at www.ajtmh.org.

Eligible children were allocated to one of four face cleaning protocols on an alternating basis as recruited with a view to including 20 children in each group. For pragmatic reasons, as multiple teams were operating in the field, eligible children in households recruited in the morning were allocated alternately to Protocols 1 and 2, and those from households recruited in the afternoons were allocated alternately to Protocols 3 and 4. All eligible children in a household were allocated to the same protocol. The protocols are given as follows: 1) the face was washed with water only (“washed with water”), 2) the face was washed with soap and water (“washed with soap”), or the face was wiped by the caregivers’ hand (“wiped with hand”) followed by handwashing for up to 30 seconds with water (Protocol 3) or water and soap (Protocol 4) (Figure 1). Protocols 3 and 4 were grouped together for analysis in this study. Face washing aimed to simulate naturalistic washes and was, thus, performed by the caregiver or the child, whichever behavior was reported as normal. Face washing technique was demonstrated and involved both hands rubbing the whole face including around the eyes for about 30 seconds, irrespective of soap use. Face wiping involved the caregiver using their hand(s) to remove visible discharge from the eyes and nose.

Children’s faces and relevant hands were examined and swabbed again (once dried) after completing the allocated protocol (immediately post-protocol), following the aforementioned procedure. Examinations and swabbing were repeated 1, 2, and 4 hours after washing protocols. Hands of caregivers of children allocated to a wiping protocol were swabbed again after handwashing (not reported). Study personnel performed all examinations and, thus, were not masked to protocol allocation.

DNA was extracted from the swabs using a commercially available kit (Biochain Blood and Serum kit, AMS Biotechnology Europe Ltd., Abingdon, UK) and tested using a previously described multiplex real-time quantitative polymerase chain reaction (qPCR) assay^6,8^ to determine the presence of *C. trachomatis*. Samples were classified as *C. trachomatis* positive if amplification of the *omcB* (chromosomal) or *pORF2* (plasmid) target was detected by qPCR in any well within 40 cycles. We performed chi-squared tests of association for the presence of discharge at each time point measured, among those with discharge before washing.

## RESULTS

We recruited 83 children aged 1–9 years (median age = 4; range, 1–9) with TF/TI from 62 households. Ocular *C. trachomatis* was detected in 11 (13%) children. Participants were from Muslim households where the main occupation was subsistence farming. Households had no access to piped water on the premises, and either an “unimproved” pit latrine or no latrine. Almost all children (80 children; 96%) reported face washing in the morning before the study, but only three (4%) had used soap. At baseline, 57 children (69%) had ocular discharge and 65 (78%) had nasal discharge.

We found significant differences in the presence of ocular discharge post-protocol (Table 1). Washing with soap was more effective at removing visible signs of ocular discharge immediately post-protocol than either washing with water (89% and 27% of discharge removed, respectively, *P* = 0.003), or wiping with a hand (42%, *P* = 0.013). No evidence was found of a difference between washing with water and wiping with a hand at removing visible signs of ocular discharge (*P* = 0.265) at this time point.

**Table 1:** Ocular and nasal discharge immediately post-protocol by the removal type, among those with ocular discharge present at baseline

Removal type	*N*	Discharge present at baseline, *N* (%)	Discharge present following protocol, *N* (% among those with discharge at baseline)	*P*-value*
Ocular discharge				
Washed with soap	17	9 (53)	1 (11)	0.013
Washed with water	19	15 (79)	11 (73)	
Wiped with hand	47	33 (70)	19 (58)	
Nasal discharge				
Washed with soap	17	11 (65)	3 (27)	
Washed with water	19	14 (74)	7 (50)	0.265
Wiped with hand	47	40 (85)	7 (18)	

“Discharge present following protocol” indicates that the washing or wiping protocol did not succeed in removing discharge from the face (or it returned immediately). The “wiped with hand” group includes children allocated to both wiping protocols, as wiping preceded protocol differences in subsequent handwashing.

* *P*-values from the χ^2^ test of association for between group comparison of the presence of post-protocol discharge, among those with discharge before washing.

**Figure 2.**
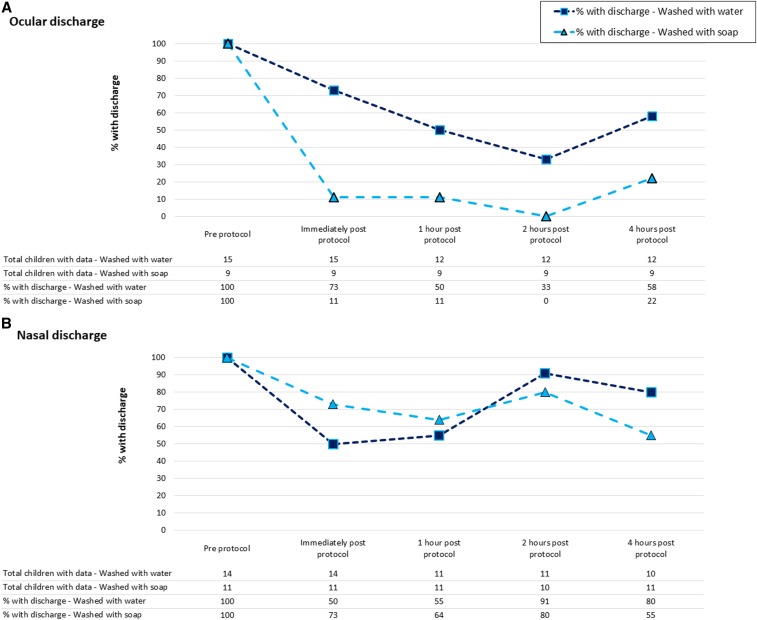
Presence of ocular and nasal discharge at five time points for children whose faces were washed with water or water and soap, among those with ocular (Graph A) or nasal (Graph B) discharge present on faces before washing. This figure appears in color at www.ajtmh.org.

Figure 2 shows how the presence of ocular and nasal discharge changed over the 4-hour follow-up. The most pronounced and sustained reduction in discharge was observed for ocular discharge in the “washed with soap” group (Figure 2A). The removal of nasal discharge by either washing method was limited and not sustained overtime (Figure 2B).

We found *C. trachomatis* on the faces of 13 children at baseline (16%), including two children without ocular *C. trachomatis* infection. Face cleaning removed *C. trachomatis* from one of three (33%) children washed with water, one of 1 (100%) child washed with soap, and three of nine (33%) children whose faces were wiped by the hands (Supplemental Table 1). Wiping transferred *C. trachomatis* to the caregiver’s hand without removing it from the child’s face on five of six occasions. No *C. trachomatis* was detected on air control swabs.

## DISCUSSION

In this study of three facial cleaning methods, we found evidence that ocular discharge was removed most effectively by washing with soap and water, and that the reduction in prevalence of ocular discharge was sustained for at least 4 hours. Comparatively, ocular discharge was removed less effectively by washing with water alone or by a caregiver wiping discharge from a child’s face with their hands. We found no significant difference between wiping and washing with water to remove ocular discharge, but wiping requires an extra step (“wiper” removing discharge transferred to their hands) to prevent onward transmission. We found no evidence that nasal discharge was removed effectively by any of the three methods.

Our finding that ocular discharge takes hours to return after washing with soap is consistent with the findings from the single other study of this type.^9^ This suggests we are most likely to interrupt transmission if face washing with soap is promoted at regular intervals throughout the day, rather than “daily,” “twice a day,” or “as frequently as necessary,” as some programs currently recommend. An outstanding question is whether face washing with soap was more effective because of a property of the soap itself (i.e., friction) or because soap use caused more thorough washing (i.e., additional rinsing to remove soap). Given that we asked people to wash their faces in the same way regardless of soap use, our results suggest that the precise mechanism may not be important as it is likely to be difficult to achieve more thorough washing with water alone.

Our results suggest that washing with water and wiping with a hand might be insufficient to remove *C. trachomatis* from the faces of children in two-thirds of cases, whereas washing with soap may be more effective, although our sample of *C. trachomatis*–positive children was small, despite all having TF/TI. These results are consistent with other published studies.^10^

The main limitations of this study were the small sample size, the nonrandom allocation of protocols, and the non-blinded nature of the intervention for researchers. The design was pragmatic, but might have introduced bias in terms of classifying the presence of discharge or consistency of the procedures (albeit we had clear standard operating procedures and training). Despite these limitations, our findings suggest that it may be pertinent to explicitly promote soap use during face washing in trachoma-endemic settings where the prevalence of ocular discharge is very high. A randomized controlled trial is needed to indicate whether face washing with soap and water can interrupt *C. trachomatis* transmission.

## Supplemental table

Supplemental materials
