# Supplementary material for: Comparison of Face Washing and Face Wiping Methods for Trachoma Control: A Pilot Study
Source: Am J Trop Med Hyg. 2020 Feb 10;102(4):740–3. doi: 10.4269/ajtmh.19-0726 (PMC7124903; doi:10.4269/ajtmh.19-0726)
Supplement: Supplementary file 1 [file tpmd190726.SD1.pdf]

The following are supplemental materials and will be published online only

Supplemental Table 1: *C. trachomatis* removal from face according to face cleaning protocol

Face cleaning protocol followed	N	*C. trachomatis* on face at baseline	Residual *C. trachomatis* detected following face cleaning (% among those with *C. trachomatis* at baseline)	*P*-value
Washed with soap	17	1	0 (0.0%)	0.321
Washed with water	19	3	1 (33.3%)	
Wiped with hand	47	9	6 (66.6%)	
**Total**	**83**	**13**	**7 (53.8%)**	
